# Antibody responses to *Plasmodium vivax* Duffy binding and Erythrocyte binding proteins predict risk of infection and are associated with protection from clinical Malaria

**DOI:** 10.1371/journal.pntd.0006987

**Published:** 2019-02-15

**Authors:** Wen-Qiang He, Ahmad Rushdi Shakri, Rukmini Bhardwaj, Camila T. França, Danielle I. Stanisic, Julie Healer, Benson Kiniboro, Leanne J. Robinson, Micheline Guillotte-Blisnick, Christèle Huon, Peter Siba, Alan Cowman, Christopher L. King, Wai-Hong Tham, Chetan E. Chitnis, Ivo Mueller

**Affiliations:** 1 Infection and Immunity Division, Walter and Eliza Hall Institute of Medical Research, Parkville, Victoria, Australia; 2 Department of Medical Biology, University of Melbourne, Melbourne, Australia; 3 Malaria Group, International Centre for Genetic Engineering and Biotechnology (ICGEB), New Delhi, India; 4 Population Health and Immunity Division, Walter and Eliza Hall Institute of Medical Research, Parkville, Victoria, Australia; 5 Insitute for Glycomics, Griffith University, Southport, Queensland, Australia; 6 Malaria Immuno-Epidemiology Unit, PNG Institute of Medical Research, Madang, Papua New Guinea; 7 Department of Parasites & Insect Vectors, Institut Pasteur, Paris, France; 8 Center for Global Health & Diseases, Case Western Reserve University, Cleveland, OH, United States of America; Johns Hopkins Bloomberg School of Public Health, UNITED STATES

## Abstract

**Background:**

The *Plasmodium vivax* Duffy Binding Protein (PvDBP) is a key target of naturally acquired immunity. However, region II of PvDBP, which contains the receptor-binding site, is highly polymorphic. The natural acquisition of antibodies to different variants of PvDBP region II (PvDBPII), including the AH, O, P and Sal1 alleles, the central region III-V (PvDBPIII-V), and *P*. *vivax* Erythrocyte Binding Protein region II (PvEBPII) and their associations with risk of clinical *P*. *vivax* malaria are not well understood.

**Methodology:**

Total IgG and IgG subclasses 1, 2, and 3 that recognize four alleles of PvDBPII (AH, O, P, and Sal1), PvDBPIII-V and PvEBPII were measured in samples collected from a cohort of 1 to 3 year old Papua New Guinean (PNG) children living in a highly endemic area of PNG. The levels of binding inhibitory antibodies (BIAbs) to PvDBPII (AH, O, and Sal1) were also tested in a subset of children. The association of presence of IgG with age, cumulative exposure (measured as the product of age and malaria infections during follow-up) and prospective risk of clinical malaria were evaluated.

**Results:**

The increase in antigen-specific total IgG, IgG1, and IgG3 with age and cumulative exposure was only observed for PvDBPII AH and PvEBPII. High levels of total IgG and predominant subclass IgG3 specific for PvDBPII AH were associated with decreased incidence of clinical *P*. *vivax* episodes (aIRR = 0.56–0.68, P≤0.001–0.021). High levels of total IgG and IgG1 to PvEBPII correlated strongly with protection against clinical vivax malaria compared with IgGs against all PvDBPII variants (aIRR = 0.38, P<0.001). Antibodies to PvDBPII AH and PvEBPII showed evidence of an additive effect, with a joint protective association of 70%.

**Conclusion:**

Antibodies to the key parasite invasion ligands PvDBPII and PvEBPII are good correlates of protection against *P*. *vivax* malaria in PNG. This further strengthens the rationale for inclusion of PvDBPII in a recombinant subunit vaccine for *P*. *vivax* malaria and highlights the need for further functional studies to determine the potential of PvEBPII as a component of a subunit vaccine for *P*. *vivax* malaria.

## Author summary

*Plasmodium vivax* is responsible for most malaria infections outside Africa, with 13.8 million vivax malaria cases reported annually worldwide. Antibodies are a key component of the host response to *P*. *vivax* infection, and their study can assist in identifying suitable vaccine candidates and serological biomarkers for malaria surveillance. The binding of *P*. *vivax* Duffy binding protein region II (PvDBPII) to the Duffy Antigen Receptor for Chemokines (DARC) is critical for *P*. *vivax* invasion of reticulocytes. Although the binding residues for DARC are highly conserved across PvDBPII, the parasite displays high sequence diversity in non-binding residues of PvDBPII. Other regions such as PvDBPIII-V are relatively conserved. Recently, sequencing of *P*. *vivax* field isolates, identified a homologous erythrocyte-binding protein (PvEBP), which harbors a domain, region II (PvEBPII), that is homologous to PvDBPII. To date, there has been limited investigation into the naturally acquired immunity to both PvDBPIII-V and PvEBPII in human populations. Using a longitudinal cohort study, we have characterized the serological response to PvDBPII, PvDBPIII-V, and PvEBPII among 1–3 years old PNG children and investigated associations with protection against clinical malaria. This study shows that both total IgG and IgG3 to the predominant PvDBPII AH allele in PNG, and total IgG and IgG1 to PvEBPII were associated with protection from *P*. *vivax* malaria.

## Introduction

*Plasmodium vivax*, which is the most widely distributed plasmodium species that infects humans [1], is considered the key challenge to malaria elimination efforts outside Africa. This is largely due to the ability of *P*. *vivax* to relapse from dormant stages in the liver [2]. These liver hypnozoites are undetectable with current diagnostic tools and treatment, which is currently limited to 8-aminoquinolines and cannot be safely prescribed to G6PD-deficient individuals [3]. Thus, additional tools to target *P*. *vivax* are urgently needed [4]. Vaccines could play an important role in the elimination of this parasite. However, as primary infections are likely to cause most of the clinical episodes and to contribute proportionately more to onward transmission than a single infection [5], it will be essential to incorporate blood-stage antigens in a candidate vaccine to reduce blood stage parasitemia and gametocytemia in breakthrough infections.

*P*. *vivax* preferentially invades young red blood cells called reticulocytes. Invasion into these cells relies on the interaction between parasite proteins and reticulocyte receptors. A well-characterized ligand-receptor pair involved in invasion is the interaction of *P*. *vivax* Duffy binding protein (PvDBP) with the Duffy Antigen Receptor for Chemokines (DARC) [6]. The virtual absence of *P*. *vivax* malaria in West Africa, where populations are generally DARC negative, highlights the central role of this pathway in *P*. *vivax* infection [7]. PvDBP is a 140 kDa type 1 integral membrane protein that consists of seven regions: a leader peptide sequence and N-terminal region (region I), conserved cysteine-rich regions (regions II and VI), central uncharacterized regions (regions III to V), and a transmembrane region followed by a cytoplasmic domain. Region II (PvDBPII) contains three-subdomain (SD) protein, with SD2 contributing key residues for binding to DARC on red blood cells (RBCs) [8, 9]. It has been proposed that binding of PvDBPII with its receptor may lead to dimerization of PvDBPII [10]. No functional role has yet been identified for central regions III to V of PvDBP. However, as they are conserved among isolates from various geographical regions [11], the potential of antibodies against these regions as correlates of protective immunity against vivax malaria deserves investigation.

In highly endemic areas of PNG, naturally acquired immunity against *P*. *vivax* controls parasite densities leading to reduced risk of clinical disease in the second and third year of life [12]. Immune responses to PvDBPII increase with age, suggesting they may play an important role in acquired immunity [13]. Strong naturally acquired humoral immunity to PvDBPII has been associated with reduced risk of high-density parasitemia in PNG children [14]. In addition, anti-PvDBPII antibodies purified from plasma from PNG individuals with the ability of blocking *P*. *vivax* invasion of reticulocytes provide the rationale of PvDBP as promising vaccine candidate [15]. However, one of concerns related to the development of PvDBP as a vaccine candidate is that sequence diversity of PvDBPII may allow the evasion of human immune responses [16, 17]. In regions of PNG with high *P*. *vivax* endemicity, the most predominant allele of PvDBPII is AH with a proportion of 26% in circulating strains [14]. In parallel to PvDBPII polymorphisms, antibody responses to PvDBPII showed strain-specific immunity to the *P*. *vivax* strains circulating in PNG [14]. When antisera were tested for the presence of functional antibodies that block PvDBPII-DARC interaction, it was found that a small proportion of individuals (<10%) were able to make high levels of binding inhibitory antibodies (BIAbs) that blocked binding of diverse strains [18]. The presence of such high levels of BIAbs was associated with protection against *P*. *vivax* infection and reduced parasite densities [18, 19]. Although PvDBPII has significant polymorphisms, the binding residues for DARC are highly conserved, which makes the development of strain-transcending BIAbs possible [18]. The reason why the development of such BIAbs is not common remains to be understood. Here, we used a functional binding inhibition assay to investigate the presence and association of anti-PvDBPII BIAbs and protection against clinical *P*. *vivax* malaria in young children in PNG of 1–3 years age.

Whole genome sequencing of Cambodian field isolates identified a second putative erythrocyte binding protein (PvEBP) with all the features of a *Plasmodium* erythrocyte-binding protein, including a N-terminal signal peptide, a Duffy-binding like domain (Region II, PvEBPII), a C-terminal cysteine-rich domain, and a transmembrane domain [20]. Although harboring all the characteristics typical of DBP superfamily member, PvEBPII seems to be distant from PvDBP in phylogeny, and no inhibition of PvEBPII-reticulocyte binding was observed by using mouse anti-PvDBPII IgG [21]. The genetic distance of PvEBPII and PvDBPII indicates that PvEBPII is not a recent gene duplication, and its apparently lower proportion of single nucleotide polymorphisms suggests that it is unlikely to be under the same level of immune selection as PvDBP [21]. In a recent screen of 38 *P*. *vivax* antigens in plasma from naturally exposed children in PNG, antibodies to both PvDBPII and PvEBPII were frequently identified among five-antigen combinations with the strongest protective effects against clinical malaria [22]. A more in-depth evaluation of the functional importance of antibody responses to variants of PvDBPII, PvDBPIII-V and PvEBPII is thus warranted.

The IgG isotype determines antibody function, and in humans, cytophilic IgG1 and IgG3 are important mediators of pathogen clearance. Numerous studies have reported that IgG subclass profiles differ among antibodies targeting different *P*. *falciparum* antigens [23–27]. The properties of the antigen appear to be one of the main determinants of the type of IgG subclass generated [25]. In addition, for some *Plasmodium* antigens, a switch from a predominant IgG1 response in young children to an increase or even predominance of IgG3 response in older individuals is a characteristic feature of natural acquisition of clinical immunity to malaria [28–30]. It remains to be confirmed if this switch is due to a history of increased exposure and/or the maturing of the immune system. Elucidating the subclasses of IgG against different PvDBPII variants and PvEBPII, and their association with clinical diseases may help better understand the importance of development of IgG subclass immunity for protection against malaria.

*Plasmodium* infection is considered to be one of the key driving forces of the evolution of the human genome. Polymorphisms in RBC proteins are particularly common in malaria endemic regions [31–33]. Gerbich deficiency is associated with the deletion of exon 3 in the glycophorin C gene (*GYPCΔex3*) [34]. Gerbich-negative erythrocytes were first identified in 1960 but are of low prevalence globally [35]. However, in some Melanesian populations from PNG, 50% of them have inherited the Gerbich phenotype [36]. Few studies have shown consistent associations between the Gerbich phenotype and *Plasmodium* infection [37–39], with one study observing a lower prevalence of *P*. *falciparum* infection among the population with this phenotype [37]. Its potential association with protection against *P*. *vivax* and its relationship with the acquisition of immunity remains unknown.

In this study, parameters of naturally acquired immunity to four variants of PvDBP (several alleles of PvDBPII and PvDBPIII-V), as well as PvEBPII were characterized in PNG children of 1–3 years of age from a 16-month longitudinal cohort study. In addition, levels of total IgG to PvDBPII, IgG subclass, and presence of anti-PvDBPII BIAbs were measured and their association with *P*. *vivax* infection, clinical episodes, and Gerbich negativity was explored.

## Material and methods

### Cohort study

Plasma samples used in the current study were collected as part of a longitudinal cohort study of young PNG children (1 to 3 years old) previously described [12]. In brief, participants were followed for up to 16 months, with visits twice/month for symptomatic illness and infection status as detected by microscopy and PCR. All *P*. *vivax* infections were genotyped, allowing for the calculation of the incidence of genetically distinct blood-stage infections acquired during follow-up (i.e. the molecular force of blood-stage infections, molFOB) [40]. Host genotyping for the presence of the *GYPCΔex3* deletion associated with the Gerbich blood group was done by PCR, as previously described [41]. Children who were homozygous for the *GYPCΔex3* deletion were considered to be Gerbich negative. Plasma samples collected at the start and at the end of the study from 224 children were used in the present study.

### Purification of PvDBPII and PvEBPII recombinant proteins

The four PvDBP variants used in this study were binding domain II from strains AH, O, P and Sal1 [14]. The recombinant PvDBPII variants were expressed in *E*. *coli*. Proteins were solubilized from inclusion bodies, purified by affinity chromatography, followed by refolding and ion exchange chromatography as per methods described earlier [42]. An 1176 bp fragment corresponding to the PvDBPIII-V region (aa 508–899) from Sal1 reference sequence was codon optimized for expression in *E*. *coli*. The protein was purified by metal affinity chromatography. Recombinant PvEBPII (aa 161–641) with a C-terminal 6-His tag was expressed as a soluble protein in *E*. *coli* SHuffle cells. Following cell lysis, the recombinant PvEBPII was purified from cell lysate as a soluble protein by metal affinity chromatography using standard procedures.

### Measurement of IgG responses by Luminex bead assay

Recombinant PvDBPII, PvDBPIII-V, and PvEBPII fragments were conjugated to Luminex Microplex microspheres as previously described [43]. To conjugate proteins to 2.5x10^6^ beads, we used 0.300 μg/mL of PvDBPII AH, 0.125 μg/mL of PvDBPII O, 0.094 μg/ mL of PvDBPII P, 0.225 μg/mL of PvDBPII Sal, 0.031 μg/mL of PvDBPIII-V and 0.250 μg/mL of PvEBPII. The Luminex multiplex bead-based antibody detection assay was performed as described elsewhere with the following modifications [29, 43]. Plasma samples were diluted 1:100 in PBS with 1% BSA and 0.05% Tween (PBT). Diluted samples were incubated with a mix of antigen-conjugated beads (0.1 uL of each bead position) (1:2) for 30 minutes under constant agitation. PE-conjugated donkey anti-human IgG Fc (0.1 mg/mL, Jackson ImmunoResearch) was used as a secondary antibody. IgG subclasses were detected using the following antibodies: mouse anti-human IgG1 hinge-PE (0.1 mg/mL, clone 4E3, Southern Biotech); mouse anti-human IgG2 Fc-PE (0.1mg/mL, clone HP6002, Southern Biotech); mouse anti-human IgG3 hinge-PE (0.1 mg/mL clone HP6050, Southern Biotech); or mouse anti-human IgG4 Fc-PE (0.1 mg/mL, clone HP6025, Southern Biotech). All these antibodies were diluted 1:100 in PBS to detect total IgG, IgG1, IgG2, IgG3, and IgG4 respectively. Beads were read on a Bio-Plex 200 reader set for 75 beads per analyte. Results were reported as median fluorescence intensity (MFI). One blank well without plasma was used for determination of the true fluorescence background. Positive controls consisted of pooled serum from immune PNG adults (>18 years) from the Madang (n = 10) and East Sepic Provinces (n = 10) who were highly exposed to malaria. Such positive controls were included in ten two-fold serial dilutions (1:50–1:25600) as pervious described [29]. Negative control sera in all assays were from the same individual, which was from the Australia Red Cross donor. The donor was anonymous resident of Melbourne, Australia with no known previous exposure to malaria.

### PvDBPII–DARC binding assays

An ELISA plate-based semi-quantitative binding assay was used to test binding of PvDBPII with DARC and estimate the binding inhibitory activity of serum as described earlier [44]. Briefly, the N-terminal 60 amino acid extracellular region of DARC was expressed as a fusion with Fc region of human IgG (nDARC-Fc), purified using protein A column and used to coat ELISA plates. Recombinant PvDBPII was incubated with nDARC-Fc coated plates in the presence of different concentrations of anti-PvDBPII serum or purified anti-PvDBPII IgG. Bound PvDBPII was detected with anti-PvDBPII rabbit sera followed by anti-rabbit IgG horse radish peroxidase (HRP)-conjugated goat antibodies. Percent binding inhibition was determined at different serum or IgG concentrations using a standard curve as previously described [44].

### Statistical analyses

Standard curves from each Luminex assay plate were used for transformation of MFIs into relative antibody units (expressed as dilution factors that range from 1.95 x 10^−5^ or 1/51200 to 0.02 or 1/50) using a five parametric logistic regression model as described previously [29]. Statistical analyses were performed using STATA version 12 (StataCorp) and R version 3.2.1 (htpp://cran.r-project.org). Spearman's rank correlation was used to assess the associations between antibody levels and age, and correlations among antibody responses against different antigens. Differences in antibody reactivity between categorical variables were assessed using Wilcoxon signed-rank sum test (for two groups) and Kruskal Wallis test (for multiple groups). Differences in proportions were evaluated by chi-square test. Antibody responses were used to predict molecular force of blood stage infection (molFOB) using a general linear model (GLM) stratified by concurrent infection status at the last visit of the study. Concurrent infection was defined as positive if PCR test was positive at the time of antibody measurement (i.e. enrollment). Antibody levels were stratified into tertiles to analyze the relationship with prospective risk of clinical *P*. *vivax* episodes (defined as axillary temperature > 37.5°C or history of fever in the preceding 48 hours with a concurrent parasitemia > 500 *P*. *vivax* /μl) and prevalence of infection diagnosed by PCR and light microscopy over the 16 months of follow-up [12]. Generalized estimating equation (GEE) with exchangeable correlation structure and semi-robust variance sandwich estimator were used and analyses were done by comparing the incidence rate ratio (IRR) of clinical malaria between the highest and lowest tertiles, and medium and low antibody levels groups. Differences in geometric mean parasitemia and incidence of clinical episodes among *GYPCΔex3* genotypes were analyzed using GEE.

To examine the effect of combining antibody responses to different antigens on the risk of clinical disease, we examined all possible combinations of 2 and 3 antigens. For this, IgG responses for each antigen were assigned a score starting from 0 to 3 for low, medium or high antibody levels (i.e. quartiles). These scores were then added up for each different combination. The scores of any combination were equally divided into three groups and used in our GEE model.

All datasets were available in the Dryad repository: https://doi.org/10.5061/dryad.n14p52b [45].

### Ethics statement

Ethics clearance was obtained from the PNG Medical Research and Advisory Committee (MRAC 05.19) and the Walter and Eliza Hall Institute (HREC 07/07) for the use of field samples. All parents/guardians of the participants signed a consent form prior to enrollment. The Melbourne control sample was obtained under ethics approval HREC 13/07.

## Results

### Total IgG levels against PvDBPII and PvEBPII in children

The pooled serum from immune PNG adults was assumed to represent the equilibrium antibody levels to all antigens achieved following repeated natural exposure. Here, we determined at enrollment the number of children who had already acquired IgG levels equivalent to >50%, >25%, >10%, >5%, or >1% of the IgG levels in adults (Table 1). Plasma from the PNG children were reactive to all four PvDBPII alleles and PvEBPII. However, total IgG levels were relatively low for the most common PvDBPII PNG variant AH, with only 25.9% and 3.6% of children achieving >5% and >25% of hyper-immune adult levels respectively. Immunogenicity of other PvDBPII alleles and PvEBPII were similar (range: 14.3–20.1%, >5% of hyper-immune adult levels) (Table 1).

**Table 1:** Total and IgG subclasses responses to PvDBP and PvEBP in Papua New Guinean children.

Protein	Antibody	Geom mean*	95% CI		No. of children (%)				
					1% of adults level	5% of adults level	10% of adults level	25% of adults level	50% of adults level
PvDBPII AH	IgG	0.26	0.22	0.32	164 (73.2)	58 (25.9)	21 (9.4)	8 (3.6)	4 (1.8)
	IgG1	0.64	0.54	0.75	220 (98.2)	123 (54.9)	59 (26.3)	21 (9.4)	7 (3.1)
	IgG3	0.33	0.26	0.43	118 (52.7)	79 (35.3)	35 (15.6)	7 (3.1)	2 (0.9)
PvDBPII O	IgG	0.44	0.37	0.53	131 (58.5)	32 (14.3)	14 (6.3)	4 (1.8)	2 (0.9)
	IgG1	1.23	1.07	1.41	185 (82.6)	62 (27.7)	29 (12.9)	8 (3.6)	3 (1.3)
PvDBPII P	IgG	0.38	0.31	0.45	130 (58.0)	45 (20.1)	17 (7.6)	7 (3.1)	3 (1.3)
	IgG1	0.99	0.84	1.17	180 (80.4)	88 (39.3)	35 (15.6)	10 (4.5)	5 (2.2)
PvDBPII Sal1	IgG	0.28	0.23	0.33	164 (73.2)	43 (19.2)	17 (7.6)	6 (2.7)	3 (1.3)
	IgG1	0.55	0.48	0.64	205 (91.5)	120 (53.6)	45 (20.1)	17 (7.6)	7 (3.1)
PvDBPIII-V	IgG	0.29	0.24	0.35	137 (61.2)	38 (17.0)	17 (7.6)	5 (2.2)	2 (0.9)
	IgG1	1.66	1.45	1.89	219 (97.8)	168 (75.0)	80 (35.7)	25 (11.2)	13 (5.8)
PvEBP	IgG	0.12	0.09	0.16	86 (38.4)	37 (16.5)	27 (12.1)	13 (5.8)	9 (4.0)
	IgG1	0.45	0.36	0.58	135 (60.3)	80 (35.7)	53 (23.7)	25 (11.2)	14 (6.3)

Abbreviation: No = number; Geom mean = geometric mean; 95% CI = 95% confidence interval.

*Values multiplied by 1000. Values in arbitrary units were interpolated from standard curves by using a 5PL logistic regression model.

**Fig 1:**
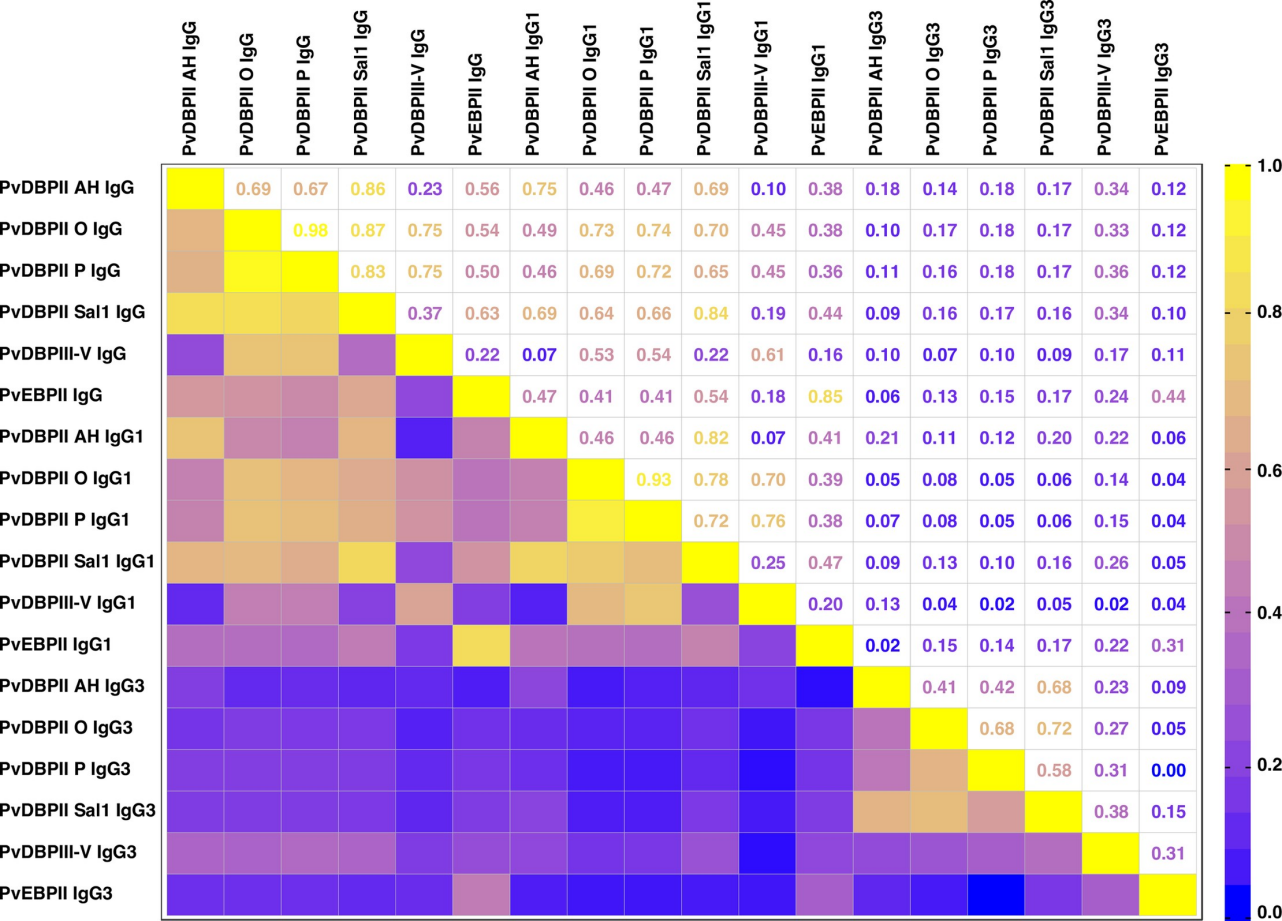
Heat map representation of the correlation between total and IgG subclass responses to PvDBPII variants and PvEBPII at enrolment. The heat map colors correspond to the Spearman correlation coefficient and range from 0 (no correlation, blue) to 1 (strong correlation, yellow). P<0.001–0.899.

Total IgG levels were strongly correlated between all proteins measured at the beginning of the cohort study (rho = 0.67–0.98, P<0.001), with the strongest correlation found between PvDBPII AH and PvDBPII O variants. Total IgG to PvEBPII shows weak to moderate correlation with different PvDBP antigens (Spearman’s rho = 0.22–0.63) (Fig 1). Similar correlation patterns were observed in the plasma samples collected at the last time point of the longitudinal study (rho = 0.22–0.99, P≤0.001) (S1 Fig).

### Immune responses in relation to infection, age, and cumulative exposure

**Fig 2:**
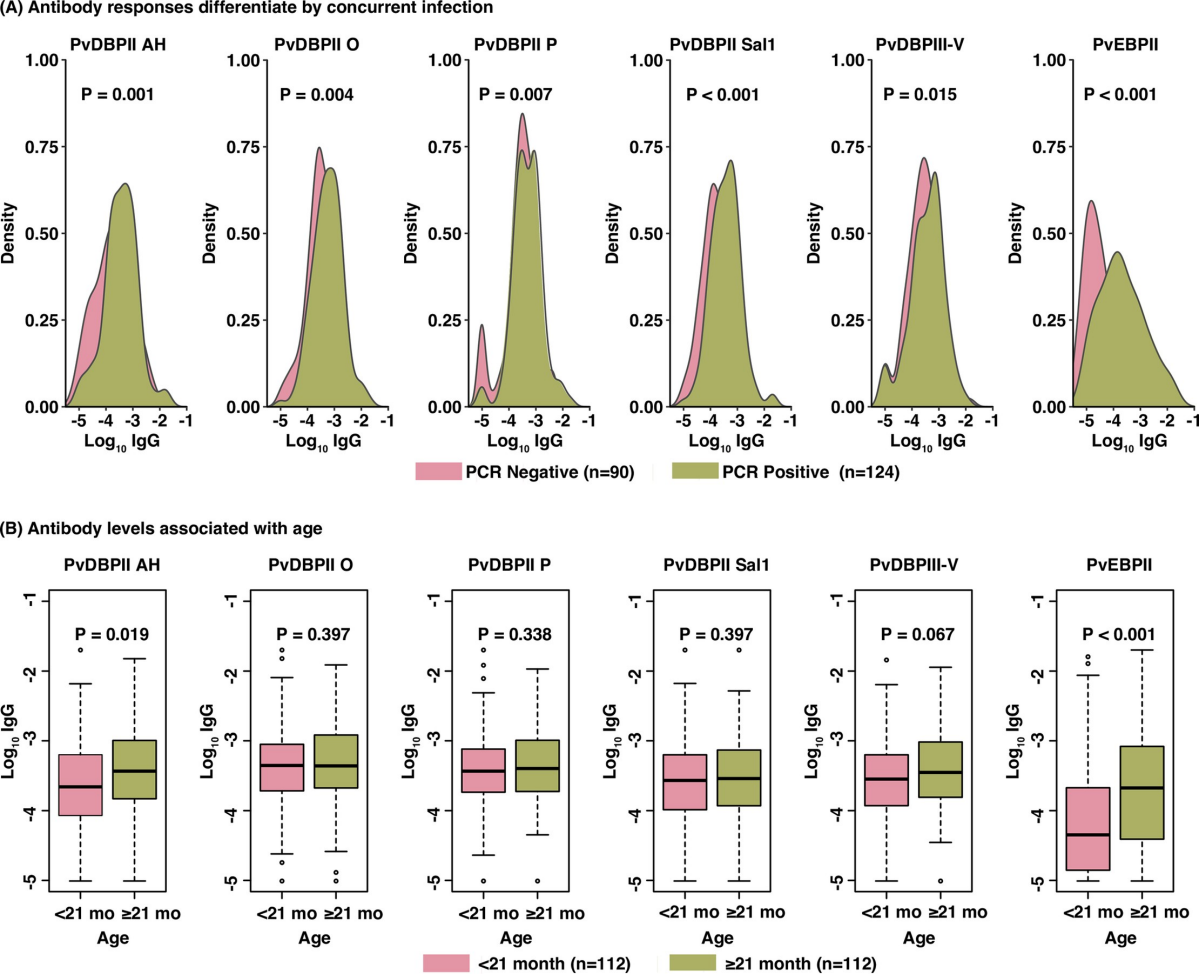
**Distribution of antibody responses by the presence of malaria infection (A) and age (B).** The X-axis represented log10 transformed antibody responses and the Y-axis represented the observed distribution of the antibody responses. Individuals without infection were shown in red and with infection in blue. Infection was determined by PCR at enrollment **(A).** Children were categorized into < 21 months of age (n = 112) and ≥ 21 months of age (n = 112) **(B)**. P values were calculated using Wilcoxon-signed rank test. P<0.05 was considered significant.

The prevalence of *P*. *vivax* infection was 55.4% (124/224) among young PNG children at enrolment, as determined by PCR (Fig 2A). Individuals with a concurrent *P*. *vivax* infection by PCR had significantly higher IgG levels (P<0.015) to all variants of PvDBPII, PvDBPIII-V and PvEBPII (Fig 2A and S1 Table), indicating that even asymptomatic *P*. *vivax* infections may boost immune responses to PvDBP and PvEBPII in settings of high *P*. *vivax* endemicity.

We examined the relationship between total IgG antibody levels with age and cumulative exposure, which was defined as the product of age and the corresponding individual molFOB [40]. Collectively, both categorical and continuous measures of antibody levels were positively correlated with age and cumulative exposure for PvDBPII AH and PvEBPII (P<0.001–0.047), and PvDBPIII-V with a borderline significance (P = 0.051), but only in children free of *P*. *vivax* infection at enrolment (Fig 2B and S1 Table). This might reflect that the acquisition of clinical immunity in this cohort of young children was mainly driven by individual exposure heterogeneity [40]. To further understand the associations of antibody responses with prevalence of infection in the longitudinal cohort study, antibody responses to PvDBPII AH and PvEBPII were found associated with increased risk of infection detected by PCR for the study period (S2 Table).

In addition, higher antibody levels were observed in the last visit for PvDBPII AH, PvDBPIII-V, and PvEBPII compared to their levels at enrolment (S2 Fig). IgG antibodies to PvDBPIII-V and PvEBPII were indeed significantly correlated with molFOB at the last visit (rho = 0.15, P≤0.020), with a borderline significance for PvDBPII AH (rho = 0.12, P = 0.070) (S2 Fig).

### IgG subclasses to PvDBP and PvEBPII

In PNG adults, IgG1 was the predominant antibody subclass for all PvDBPII proteins (S3 Fig). IgG2 and IgG3 were the subdominant antibody subclasses, with substantially higher amounts of IgG3 detected against the most common PvDBPII AH variant than the other strains. Balanced responses with detectable amounts of IgG1 and IgG3 antibodies were found for PvDBPIII-V and PvEBPII. No detectable levels of IgG4 were observed for any of the antigens tested (S3 Fig).

Between 27.7–75.0% of the children had levels of IgG1 against PvDBPII and PvEBPII that were >5% of the IgG1 levels observed in adults to all antigens tested. However, only a small subset of children (range: 3.6–11.2%) had IgG1 levels exceeding 25% of adult levels (Table 1). In contrast to what was observed in adults, children showed a strong IgG1 predominance among antibodies to PvEBPII. Detectable levels of IgG3 were observed for PvDBPII AH and PvEBPII, both in much lower levels than IgG1, suggesting that acquisition of IgG1 was faster than IgG3 for both PvEBPII and PvDBP antigens. Polarization from IgG1 towards IgG3 was only identified for PvDBPII AH, as suggested by the decreasing ratio of IgG1/IgG3 with increase in age (rho = -0.24, P<0.001). No detectable levels of IgG2 and IgG4 were observed for any of the antigens tested among these children.

PCR-positive children had increased IgG1 for PvDBPII AH, PvDBPII O, and PvEBPII (S1 Table). Consistent with the patterns observed for total IgG, significant increase in IgG1 with age and cumulative exposure were identified for PvDBPII AH and PvEBPII in PCR negative children (rho = 0.25–0.42, P≤0.013) but not in those with concurrent infections (S1 Table). Increases in IgG3 against PvDBPII AH and PvEBPII were significantly associated with age and cumulative exposure (rho = 0.19–0.39, P≤0.044) (S1 Table).

### Total IgG to PvDBP and PvEBPII reduces the risk of clinical malaria

**Fig 3:**
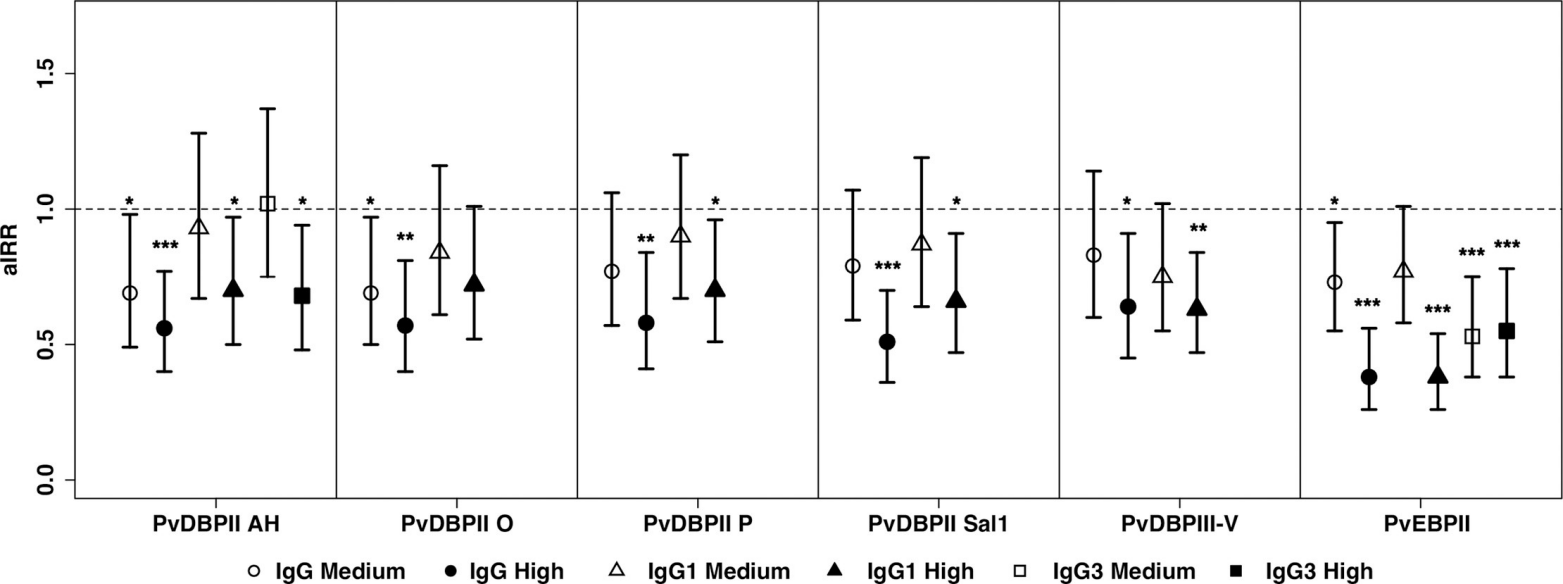
Association between total and IgG subclasses to four PvDBPII variants, PvEBPII and protection against clinical malaria (density>500 parasite/ul) in 224 young Papua New Guinean children. Data were plotted as exposure (molFOB), age, season and village of residency adjusted incidence rate ratios and 95% confidence intervals. Incidence rate ratios, 95% confidence intervals and P-values from GEE models. P<0.05 were deemed significant. ^*^ denotes P<0.05, ** denotes P<0.01, *** denotes P<0.001.

Children with medium and high levels of IgG to PvDBPII AH allele had 31% and 44% reduction in the risk of a *P*. *vivax* clinical episodes, respectively, compared to children with low antibody levels (adjusted incidence risk ratio medium versus low antibody levels (aIRR_M_ 0.69, 95% CI: 0.49–0.98, P = 0.037); high versus low antibody levels (aIRR_H_ 0.56, 95% CI, 0.40–0.77, P <0.001) (Fig 3 and S3 Table). IgG to PvDBPII O showed similar, but slightly lower significant protective association (Fig 3 and S3 Table). Therefore, total IgG responses to PvDBPII AH and PvDBPII O may be biomarkers of protective immunity. Only children with high IgG antibody levels to PvDBPII P, PvDBPII Sal1 and PvDBPIII-V had a significant reduction in the risk of *P*. *vivax* malaria. Antibodies to PvEBPII correlated with stronger protection than all variants of PvDBPII (aIRR_M_ = 0.73, P = 0.022; aIRR_H_ = 0.26, P<0.001, Fig 3 and S3 Table) and in a multivariate model, only antibodies to PvEBPII remained independently associated with protection against clinical malaria (S3 Table).

We further examined the possible effect of combining antibody levels against PvDBPs and PvEBPII on the risk of clinical disease. Combinations of PvDBPII AH and PvEBPII showed evidence of an increased protective effect (aIRR = 0.30, 95% CI, 0.19–0.46, P<0.001, S4 Table**)**. Combinations of 3 antigens did not show an additional increase in protection (S4 Table).

### IgG subclass responses to PvDBP and PvEBPII and risk of clinical disease

For all of the antigens tested, with the exception of PvDBPII O, high levels of IgG1 were associated with decreased risk of clinical malaria in the adjusted models (aIRR_H_ = 0.38–0.70, P ≤ 0.027) (Fig 3 and S3 Table). For IgG3 responses, the analysis was restricted to PvDBPII AH and PvEBPII as detectable antibody levels were only observed for them. Both PvDBPII AH and PvEBPII responses also showed a protective effect (aIRR_H_ = 0.55–0.68, P≤0.021). In a multivariate model incorporating IgG1 and IgG3 for all antigens, only IgG3 to PvDBPII AH and IgG1 to PvEBPII remained associated with clinical protection (aIRR = 0.38–0.63, P ≤ 0.006) (S3 Table).

### Anti-PvDBPII binding inhibitory antibodies against diverse *P*. *vivax* strains

Plasma obtained at first (n = 8) and last visit (n = 8) exhibited substantial binding inhibitory antibodies against diverse PvDBPII alleles (Table 2). Binding inhibitory antibodies against the three PvDBPII variants was also significantly correlated (P<0.001) with the highest correlation observed between the two most prevalent alleles PvDBPII AH and PvDBPII O (rho = 0.66, P<0.001).

**Table 2:** Blocking activity of antibodies against different PvDBPII alleles.

	Enrolment (n = 168)				End of follow-up (n = 162)			
	AH	O	Sal 1	All 3	AH	O	Sal 1	All 3
Median OD	37	36.4	34.9		41.3	42.3	38.6	
IQR	[23.4, 50.2]	[25.0, 45.5]	[24.0, 47.2]		[28.6, 52.6]	[32.0, 49.8]	[29.4, 45.9]	
Min–max	0–100	0–100	0–99.4		0–100	0–100	0–100	
**≥80%**								
n positive	6	6	8	6	7	6	7	6
% positive	3.57%	3.57%	4.76%	3.57%	4.32%	3.70%	4.32%	3.70%
**60–79%**								
n positive	12	9	10	8	11	10	10	8
% positive	7.14%	5.36%	5.95%	4.76%	6.79%	6.17%	6.17%	4.94%

Abbreviations: IQR = interquartile range.

Twelve children (7.14% of 168 tested) had ≥60% blocking activity for at least one variant of PvDBPII. Eight children (4.76%) had high levels (≥80% blocking activity) of inhibition to one variant, six of which showed high blocking activity against all three variants. Children with concurrent *P*. *vivax* infections showed moderate-high blocking activity (>60% blocking against all three alleles, PCR positive: 13.3% vs. PCR negative: 3.5%, P = 0.027). Although median blocking activity did not vary with age (P>0.19), five of the six children with high levels of inhibitory, strain-transcending antibodies were older than 21 months of age (P = 0.115).

Blocking activity in plasma samples collected at the end of follow-up was very similar to the start of the study (Table 2). After 16 months of additional exposure, concurrent *P*. *vivax* infections were no longer associated with an increase in blocking activity. Only three children (1.9%) had high blocking antibodies (≥80%) against all three variants at both time points. Among the three children with constant high blocking activity, two (66.7%) were homozygous for the Gerbich blood group (i.e. *GYPC*Δ*ex3*) compared to 14 (10.5%) in those with lower or no blocking activity (P = 0.036).

When assessing the association between the ability of antibodies to block PvDBPII binding to red blood cells and prospective risk of *P*. *vivax* malaria, children with high blocking ability against AH (IRR = 0.44, P = 0.059), O (IRR = 0.52, P = 0.119), Sal1 (IRR = 0.52, P = 0.081), or all three alleles combined (IRR = 0.45, P = 0.083) at enrolment showed a tendency for a reduced incidence of *P*. *vivax* episodes of any density (Table 3). These effects were almost entirely due to the three children with high strain-transcending blocking activity at both enrolment and end of follow-up (IRR = 0.16, 95% CI, 0.03–1.04, P = 0.055).

**Table 3:** Association of anti-PvDBPII binding inhibitory antibodies and protection against subsequent *P*. *vivax* malaria.

Antigens	Levels of inhibition	Pv any density				Pv > 500/μl			
		IRR	95% CI		P value	IRR	95% CI		P value
AH	60–79%	1.08	0.7	1.8	0.761	0.81	0.43	1.54	0.518
	≥80%	0.44	0.2	1.0	0.059	0.53	0.19	1.54	0.245
O	60–79%	1.02	0.6	1.8	0.951	0.93	0.47	1.85	0.844
	≥80%	0.52	0.2	1.2	0.119	0.71	0.28	1.8	0.47
Sal 1	60–79%	1.07	0.6	1.8	0.789	1.17	0.62	2.2	0.636
	≥80%	0.54	0.3	1.1	0.081	0.56	0.23	1.36	0.201
All 3	60–79%	1.04	0.6	1.9	0.901	1.58	0.63	3.92	0.326
	≥80%	0.45	0.2	1.1	0.083	0.42	0.1	1.75	0.234

Abbreviation: IRR = incidence rate ratio; 95% CI = 95% confidence interval. IRR is for responders of high level and medium level versus those of low levels, 95% confidence intervals and P values are obtained from GEE models. P values <0.05 were deemed significant.

Neither blocking nor total IgG antibodies to any of the *P*. *vivax* proteins showed any protective association with the risk of *P*. *falciparum* clinical episodes, but all children with high levels of total IgG were associated with increased episodes of clinical *P*. *falciparum* malaria, suggesting antibodies against PvDBP and PvEBP were correlates of increased risk of *P*. *falciparum* exposure (S5 Table).

### Gerbich-negativity is associated with higher antibody responses, lower *P*. *vivax* parasitemia, and lower risk of clinical disease

In this study, 29 children harbored Gerbich phenotype caused by double deletion of exon 3 of *GYPC* gene, 111 of them with single deletion of the same region named as heterozygote and other 84 were without any deletion called wild-type. Children with Gerbich phenotype had a reduced risk of malaria episodes in comparison to those with wild-type, and the strength of this relationship increased with increasing parasite densities (aIRR = 0.69, 95%CI = 0.41–1.01, P = 0.040, for *P*. *vivax* >500 parasites /μL; aIRR = 0.53, 95%CI = 0.28–1.00, P = 0.050 for >2,000 parasites /μL; aIRR = 0.40, 95%CI = 0.17–0.94, P = 0.036 for >10,000 parasites /μL; Table 4). Similarly, the geometric mean parasitemia was significantly lower in children with homozygous Gerbich phenotype than those with wild-type (P = 0.003).

**Table 4:** Association between Gerbich negativity and incidence of clinical malaria during follow-up in 1–3 year PNG children.

Clinical malaria	Wild Type (n = 84)			Heterozygote (wt/Δ3, n = 111)			Gerbich negativity (Δ3/Δ3, n = 29)					
	Events	PYAR	Incidence	Events	PYAR	Incidence	Events	PYAR	Incidence	aIRR*	95% CI	P value
All episodes	395	102.2	3.86	479	135	3.55	122	35.5	3.44	0.82	0.65–1.03	0.088
All *Pf* episode	228	102.2	2.23	259	135	1.92	75	35.5	2.11	0.81	0.60–1.08	0.154
*Pf*>2500/ul	169	102.2	1.65	195	135	1.44	56	35.5	1.58	0.84	0.58–1.21	0.343
All *Pv* episodes	220	102.2	2.15	257	135	1.90	54	35.5	1.52	0.69	0.48–0.98	**0.040**
*Pv*>500/ul	134	102.2	1.31	168	135	1.24	34	35.5	0.96	0.64	0.41–1.01	0.054
*Pv*>2000/ul	93	102.2	0.91	114	135	0.84	21	35.5	0.59	0.53	0.28–1.00	**0.050**
*Pv*>10000/ul	40	102.2	0.39	44	135	0.33	6	35.5	0.17	0.40	0.17–0.94	**0.036**

Abbreviation: PYAR = person year at risk; aIRR = adjusted incidence rate ratio; Pf = *Plasmodium falciparum*; Pv = *Plasmodium vivax*.

*aIRR were shown the comparison between homozygotes and wild-type group by applying GEE models with analysis adjusted for the following potential confounders: the village of residence, age, the season of recruitment, and force of infection. P<0.05 were deemed significant.

**Fig 4:**
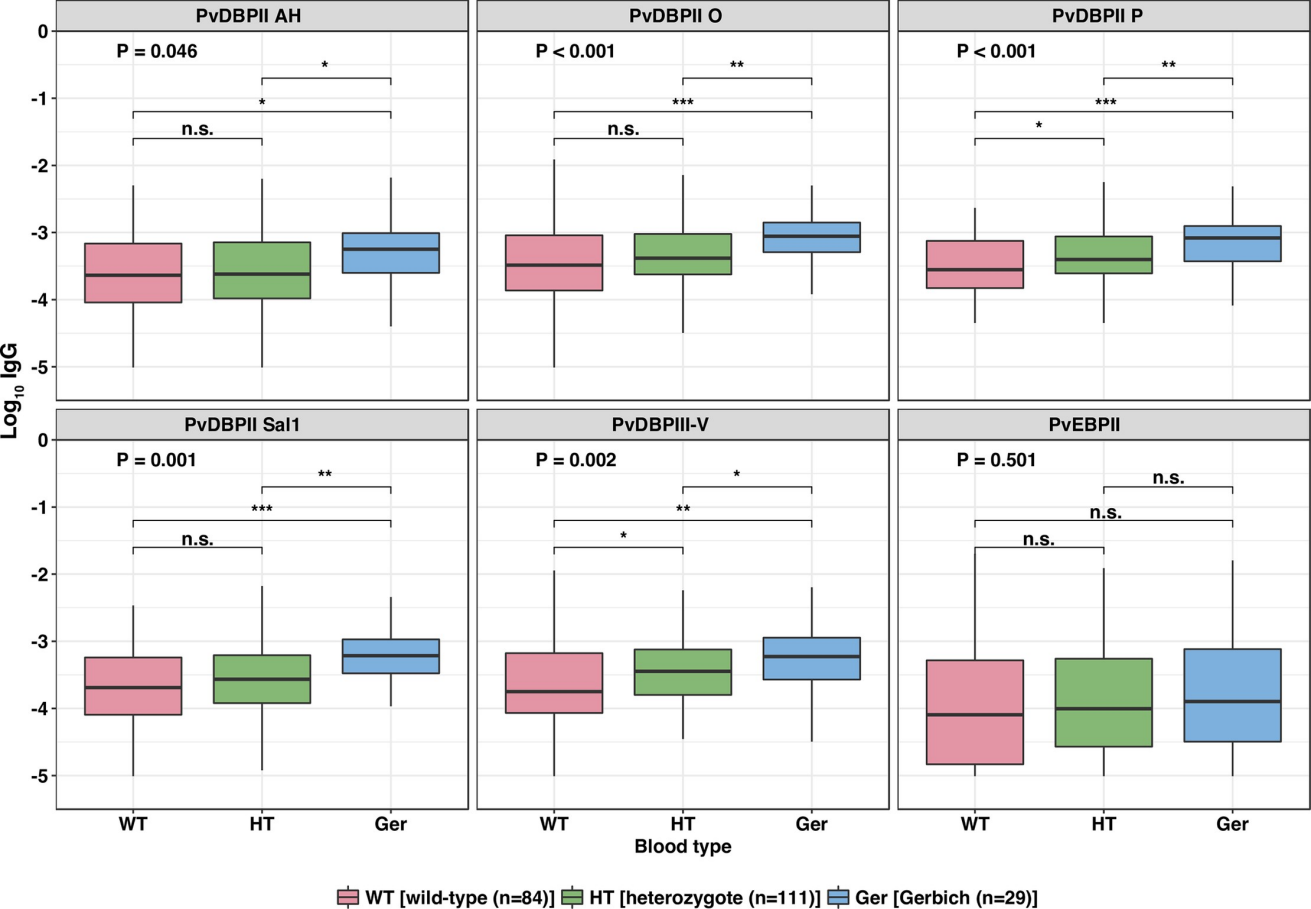
Gerbich blood type correlates with stronger antibody responses for PvDBPII variants. The overall differences among three groups by red blood cell phenotype were compared using Kruskal-Wallis one-way analysis, and individual comparison of each two groups was tested by Wilcoxon signed-rank sum method, star represents the comparison to wild-type group. P<0.05 were deemed significant. ^*^ denotes P<0.05, ** denotes P<0.01, *** denotes P<0.001.

Children with wild-type phenotype had the lowest levels of antibodies against all PvDBP variants, while homozygotes had the highest levels to almost all antigens (P<0.011–0.046), except for PvEBPII (P = 0.501) (Fig 4). These results suggested that *GYPCΔex3* may contribute to the acquisition of antibodies to PvDBP but not to PvEBPII in PNG children.

## Discussion

This study confirmed that total IgG and infrequently detected BIAbs against PvDBPII were associated with an overall lower incidence rate of clinical vivax malaria in young children who were developing clinical immunity to *P*. *vivax*. Antibody responses against PvDBPII were higher in those Gerbich-negative, a common red blood cell polymorphism within the East Sepik region of PNG. We also observed a strong association with protection for total IgG and IgG1 antibodies to PvEBPII, but no difference by Gerbich phenotype.

In this study, only 4.8% (8/168) of the young children aged 1 to 3 years had acquired high levels of BIAbs (> 80% binding inhibition) against at least one PvDBPII allele, with six children exhibiting binding-inhibitory antibodies against diverse strains, while none of them had obtained levels higher than of 90% of BIAbs against any PvDBPII allele. An earlier study among school-age children (5–9 years) in PNG identified 9% of children with BIAbs >90% to PvDBPII [18] and in the Brazilian Amazon, 26.6% and 20.5% of the residents of all age groups presented >80% and >90% BIAbs activity to PvDBPII [19]. In these two studies [18, 19], high anti-PvDBPII BIAbs activity blocked diverse strains. The overall lower detection of high-levels PvDBPII BIAbs in our study indicate that acquisition of BIAbs is at least partially related to the increase in life-time malaria exposure. However, the observation that BIAbs are not common even in adult population with high levels of *P*. *vivax* exposure indicates that these functional blocking antibodies are difficult to acquire under conditions of natural exposure. Nevertheless, since the target epitope of BIAbs to PvDBPII was conserved [46], once PvDBPII BIAbs are acquired, they may provide strain transcending binding inhibitory activity, even in young children with limited and developing clinical immunity.

In contrast to the DARC conserved binding residues, polymorphisms thought to be associated with immune evasion [10] are common throughout nonfunctional regions of PvDBPII distal to the binding site of DARC. Our study and previous reports show that these regions of PvDBPII are exposed to the immune system resulting in observable immune responses to PvDBPII generated at a young age, even in our cohort of young children with limited immunity [14, 18]. However, antibody responses to these polymorphic PvDBPII regions are likely to be strain-specific [14] and potentially not functionally associated with clinical protection [18, 19]. In this young cohort of PNG children, IgG to the dominant variants PvDBPII AH and O were both more prevalent and strongly associated with protection against clinical vivax-malaria in the adjusted models. As such, total IgG responses to these PvDBPII variants may serve as biomarkers for protective immunity.

Regions II and VI of PvDBP are cysteine-rich domains and under immune selection, while regions III to V do not have a known function [8]. Nevertheless, one study in *P*. *falciparum* showed that antibodies to regions III-V of PfEBA175 and PfEBA140 could inhibit *P*. *falciparum* invasion [47]. Our study showed that antibodies to region III-V predicted protection from vivax clinical malaria. However, antibody titers to region III-V were significantly correlated to antibodies against some PvDBPII variants and did not retain a significant association with protection in the multivariate models. Future functional studies will be required to investigate the potential functional role of antibodies to region III-V of PvDBP.

Among all antigens tested, the strongest association of protection from vivax malaria was identified for PvEBPII. In one previous study, PvEBPII was characterized as a functionally and antigenically distinct *P*. *vivax* ligand with a stronger binding preference for Duffy-positive than Duffy-negative reticulocytes *in vitro* [21], suggesting that although antigenically distinct from PvDBP, it may function as a redundant invasion pathway when immune activity blocks the principal PvDBPII-DARC pathway. The relatively low correlation between antibody responses to PvDBP and PvEBPII indicates co-acquisition of antibodies to both antigens, rather than cross-reactivity between them. Combination of PvDBPII AH and PvEBPII immune responses offered a further increase in protection against clinical vivax-malaria and thus supports the inclusion of these two antigenically distinct ligands in a combination vaccine. PvEBPII should be further investigated as a potential vaccine candidate, and the efficacy of anti-PvEBP antibodies will need to be confirmed in functional assays.

Some polarization of IgG1 towards IgG3 was only identified for PvDBPII AH, as similar switch towards a balanced IgG1/IgG3 response was observed in the narrow age range of our study population. The possible reason for this might be related to the higher circulation of AH strain in this setting, thus young children might be exposed to the AH strain enabling them to acquire higher IgG3 to AH. Apart from the possible higher exposure, by comparing with other alleles included in this manuscript, AH was shown to have two special mutations K371E and K386Q, both of which were mapped to SD2. As most of the PvDBPII polymorphism are in this subdomain, our results suggest that these two mutations were important for immune reactivity to PvDBPII AH. Whereas, after a longer period of immune exposure to other strains, including O, P and Sal1, significantly higher levels of IgG3 to them were also observed in the adults. Consistent with previous reports [25, 28], these results indicated that IgG3 switching may be driven by the nature of the antigen and influenced by exposure and maturation of the immune system. In multivariate models of isotype-specific responses, for IgG3 against PvDBPII AH and high IgG1 to PvEBPII were significantly associated with a reduced risk of clinical vivax-malaria. It remains to be confirmed whether IgG1 and IgG3 antibodies target different epitopes and/or differ in their functionality or if they simply differ in their utility as correlates of risk of future infection or protection against vivax malaria.

In addition to antibody-mediated protection against vivax malaria, it is believed that specific red blood cell polymorphisms can induce resistance to clinical malaria [33]. There is limited evidence that individuals with Gerbich negativity may have a lower risk of *P*. *falciparum* and/or *P*. *vivax* infection [37]. Our longitudinal study now provides the first indication for a protective role of the Gerbich phenotype with reduction of *P*. *vivax* malaria among young children aged 1 to 3 years with limited clinical immunity. However, Gerbich negativity was not associated with significant protection against blood-stage infection in school-aged children (5–14 years) from another PNG longitudinal cohort study [39]. It is assumed that the acquired, clinical immunity among older semi-immune children and immune adults may mask the protective effects of specific genotypes against uncomplicated malaria infection [39]. In this study, a higher proportion of Gerbich homozygotes was found among children with high titers of PvDBPII-specific BIAbs. This was consistent with a previous observation that children with the South-East Asian ovalocytosis (SAO, caused the *SLC4A1Δ27* deletion in the human Band 3) were 3.3 times more likely than non-SAO children to have high levels of PvDBPII-specific BIAbs [48]. The mechanism by which Gerbich affects anti-PvDBP antibody responses is unknown and will require further investigation.

In summary, our study highlights the association of total antibody and BIAbs to PvDBPII variants with lower risk of clinical *P*. *vivax* malaria episodes in PNG children and further strengthens the rationale for PvDBPII as a potential vaccine antigen. Interestingly, both naturally acquired immunity to PvDBPII and Gerbich homozygosity showed significant protection against *P*. *vivax*. Antibodies to PvEBPII were more strongly association with clinical protection against *P*. *vivax* malaria in these young children. Further studies will be needed to clarify the mechanism of protection afforded by Gerbich and the importance of PvEBPII in *P*. *vivax* reticulocyte invasion and immune protection.

## Supporting information

S1 Checklist STROBE statement—Checklist of items that should be included in reports of cohort studies. (DOC)

S1 Fig Heat map representation of the correlation of antibody responses to PvDBP variants and PvEBPII at enrollment and end of the cohort study. The heat map colors correspond to the Spearman’s correlation coefficient, ranging from 0 (no correlation, blue) to 1 (strong correlation, yellow). P<0.001–0.152.
(TIF)

S2 Fig The relationship of antibody responses to PvDBP variants and PvEBPII and individual exposure. **(A)** The comparison of antibody levels between enrolment and end of the cohort study. Red represents the first visit and blue represents the last visit. P values were calculated using Wilcoxon signed-rank sum method. P values <0.05 were considered significant. **(B)** Association of antibody levels from the last visit of the study and the molecular force of blood stage infection (molFOB). The blue lines show the association between antibody responses and molFOB predicted by linear regression models. The shaded regions depict the variation in the data (95% prediction interval). X-axis: molFOB, y-axis: total IgG antibody responses for each antigen. P values are from general linear model. P values and were deemed significant if <0.05.
(TIF)

S3 Fig IgG subclasses response patterns among PNG adults and young children. Antibody levels of crude mean fluorescence intensity (MFI) were log10 transformed. Solid lines represent antibody levels among adults in a two-fold serial dilution starting from 1/50. Only the median antibody levels among children for each subclass (IgG1, IgG2 and IgG3) were presented by dashed lines.
(TIF)

S4 Fig The distribution of crude antibody responses of total IgG, IgG1, IgG2 and IgG3 to PvDBP and PvEBPII at enrollment. Antibody levels of crude mean fluorescence intensity (MFI) were shown in X axis and count of each level were represented in Y axis. Antibody levels of total IgG, IgG1, IgG2 and IgG3 were depicted in pink, yellow, blue, and light slate blue respectively.
(TIF)

S1 Table Associations between IgG and IgG subclasses to PvDBP and PvEBPII with measures of concurrent and cumulative exposure. (XLSX)

S2 Table Association between antibodies and prevalence of *P. vivax* infection diagnosed by PCR. (XLSX)

S3 Table Association between antibodies and risk of clinical *P. vivax* malaria. (XLSX)

S4 Table The association of combination of antibody responses and risk of *P. vivax* malaria. (XLSX)

S5 Table Association between antibodies and risk of clinical *P. falciparum* malaria. (XLSX)

## References

[1] Howes RE, Battle KE, Mendis KN, Smith DL, Cibulskis RE, Baird JK, et al Global Epidemiology of Plasmodium vivax. The American journal of tropical medicine and hygiene. 2016;95(6 Suppl):15–34. 10.4269/ajtmh.16-0141 27402513 PMC5198891

[2] Mueller I, Galinski MR, Baird JK, Carlton JM, Kochar DK, Alonso PL, et al Key gaps in the knowledge of Plasmodium vivax, a neglected human malaria parasite. Lancet Infect Dis. 2009;9(9):555–66. 10.1016/S1473-3099(09)70177-X 19695492

[3] Uthman OA, Graves PM, Saunders R, Gelband H, Richardson M, Garner P. Safety of primaquine given to people with G6PD deficiency: systematic review of prospective studies. Malar J. 2017;16(1):346 10.1186/s12936-017-1989-3 28830424 PMC5568268

[4] Baird K. Origins and implications of neglect of G6PD deficiency and primaquine toxicity in Plasmodium vivax malaria. Pathogens and global health. 2015;109(3):93–106. 10.1179/2047773215Y.0000000016 25943156 PMC4455359

[5] Wampfler R, Hofmann NE, Karl S, Betuela I, Kinboro B, Lorry L, et al Effects of liver-stage clearance by Primaquine on gametocyte carriage of Plasmodium vivax and P. falciparum. PLOS Negl Trop Dis. 2017;11(7):e0005753 10.1371/journal.pntd.0005753 28732068 PMC5540608

[6] Chitnis CE, Miller LH. Identification of the erythrocyte binding domains of Plasmodium vivax and Plasmodium knowlesi proteins involved in erythrocyte invasion. The Journal of experimental medicine. 1994;180(2):497–506. 8046329 10.1084/jem.180.2.497 PMC2191600

[7] Miller LH, Mason SJ, Clyde DF, McGinniss MH. The resistance factor to Plasmodium vivax in blacks. The Duffy-blood-group genotype, FyFy. N Engl J Med. 1976;295(6):302–4. 10.1056/NEJM197608052950602 778616

[8] Adams JH, Sim BK, Dolan SA, Fang X, Kaslow DC, Miller LH. A family of erythrocyte binding proteins of malaria parasites. Proceedings of the National Academy of Sciences. 1992;89(15):7085–9. 10.1073/pnas.89.15.7085 PMC49650 1496004

[9] Batchelor JD, Malpede BM, Omattage NS, DeKoster GT, Henzler-Wildman KA, Tolia NH. Red blood cell invasion by Plasmodium vivax: structural basis for DBP engagement of DARC. PLoS Pathog. 2014;10(1):e1003869 10.1371/journal.ppat.1003869 24415938 PMC3887093

[10] Batchelor JD, Zahm JA, Tolia NH. Dimerization of Plasmodium vivax DBP is induced upon receptor binding and drives recognition of DARC. Nature structural & molecular biology. 2011;18(8):908–14. 10.1038/nsmb.2088 PMC3150435 21743458

[11] Martinez P, Suarez CF, Cardenas PP, Patarroyo MA. Plasmodium vivax Duffy binding protein: a modular evolutionary proposal. Parasitology. 2004;128(Pt 4):353–66. 15151140 10.1017/s0031182003004773

[12] Lin E, Kiniboro B, Gray L, Dobbie S, Robinson L, Laumaea A, et al Differential patterns of infection and disease with P. falciparum and P. vivax in young Papua New Guinean children. PLOS ONE. 2010;5(2):e9047 10.1371/journal.pone.0009047 20140220 PMC2816213

[13] Cole-Tobian JL, Cortés A, Baisor M, Kastens W, Xainli J, Bockarie M, et al Age-Acquired Immunity to a Plasmodium vivax Invasion Ligand, the Duffy Binding Protein. J Infect Dis. 2002;186(4):531–9. 10.1086/341776 12195381

[14] Cole-Tobian JL, Michon P, Biasor M, Richards JS, Beeson JG, Mueller I, et al Strain-specific duffy binding protein antibodies correlate with protection against infection with homologous compared to heterologous plasmodium vivax strains in Papua New Guinean children. Infection and Immunity. 2009;77(9):4009–17. 10.1128/IAI.00158-09 19564376 PMC2737995

[15] Grimberg BT, Udomsangpetch R, Xainli J, McHenry A, Panichakul T, Sattabongkot J, et al Plasmodium vivax invasion of human erythrocytes inhibited by antibodies directed against the Duffy binding protein. PLoS Med. 2007;4(12):e337 10.1371/journal.pmed.0040337 18092885 PMC2140086

[16] Ampudia E, Patarroyo MA, Patarroyo ME, Murillo LA. Genetic polymorphism of the Duffy receptor binding domain of Plasmodium vivax in Colombian wild isolates. Molecular and biochemical parasitology. 1996;78(1–2):269–72. 8813697 10.1016/s0166-6851(96)02611-4

[17] Xainli J, Adams JH, King CL. The erythrocyte binding motif of plasmodium vivax duffy binding protein is highly polymorphic and functionally conserved in isolates from Papua New Guinea. Molecular and biochemical parasitology. 2000;111(2):253–60. 11163434 10.1016/s0166-6851(00)00315-7

[18] King CL, Michon P, Shakri AR, Marcotty A, Stanisic D, Zimmerman PA, et al Naturally acquired Duffy-binding protein-specific binding inhibitory antibodies confer protection from blood-stage Plasmodium vivax infection. Proc Natl Acad Sci USA. 2008;105(24):8363–8. 10.1073/pnas.0800371105 18523022 PMC2448842

[19] Nicolete VC, Frischmann S, Barbosa S, King CL, Ferreira MU. Naturally Acquired Binding-Inhibitory Antibodies to Plasmodium vivax Duffy Binding Protein and Clinical Immunity to Malaria in Rural Amazonians. J Infect Dis. 2016;214(10):1539–46. 10.1093/infdis/jiw407 27578850 PMC5091372

[20] Hester J, Chan ER, Menard D, Mercereau-Puijalon O, Barnwell J, Zimmerman PA, et al De novo assembly of a field isolate genome reveals novel Plasmodium vivax erythrocyte invasion genes. PLOS Negl Trop Dis. 2013;7(12):e2569 10.1371/journal.pntd.0002569 24340114 PMC3854868

[21] Ntumngia FB, Thomson-Luque R, Torres Lde M, Gunalan K, Carvalho LH, Adams JH. A Novel Erythrocyte Binding Protein of Plasmodium vivax Suggests an Alternate Invasion Pathway into Duffy-Positive Reticulocytes. MBio. 2016;7(4). 10.1128/mBio.01261-16 PMC4999553 27555313

[22] Franca CT, White MT, He WQ, Hostetler JB, Brewster J, Frato G, et al Identification of highly-protective combinations of Plasmodium vivax recombinant proteins for vaccine development. eLife. 2017;6. 10.7554/eLife.28673 PMC5655538 28949293

[23] Taylor RR, Smith DB, Robinson VJ, McBride JS, Riley EM. Human antibody response to Plasmodium falciparum merozoite surface protein 2 is serogroup specific and predominantly of the immunoglobulin G3 subclass. Infect Immun. 1995;63(11):4382–8. 7591074 10.1128/iai.63.11.4382-4388.1995 PMC173623

[24] Rzepczyk CM, Hale K, Woodroffe N, Bobogare A, Csurhes P, Ishii A, et al Humoral immune responses of Solomon Islanders to the merozoite surface antigen 2 of Plasmodium falciparum show pronounced skewing towards antibodies of the immunoglobulin G3 subclass. Infect Immun. 1997;65(3):1098–100. 9038322 10.1128/iai.65.3.1098-1100.1997 PMC175094

[25] Stanisic DI, Fowkes FJI, Koinari M, Javati S, Lin E, Kiniboro B, et al Acquisition of antibodies against Plasmodium falciparum merozoites and malaria immunity in young children and the influence of age, force of infection, and magnitude of response. Infection and Immunity. 2015;83(2):646–60. 10.1128/IAI.02398-14 25422270 PMC4294228

[26] Branch OH, Oloo AJ, Nahlen BL, Kaslow D, Lal AA. Anti-merozoite surface protein-1 19-kDa IgG in mother-infant pairs naturally exposed to Plasmodium falciparum: subclass analysis with age, exposure to asexual parasitemia, and protection against malaria. V. The Asembo Bay Cohort Project. J Infect Dis. 2000;181(5):1746–52. 10.1086/315424 10823777

[27] Egan AF, Chappel JA, Burghaus PA, Morris JS, McBride JS, Holder AA, et al Serum antibodies from malaria-exposed people recognize conserved epitopes formed by the two epidermal growth factor motifs of MSP1(19), the carboxy-terminal fragment of the major merozoite surface protein of Plasmodium falciparum. Infect Immun. 1995;63(2):456–66. 7822010 10.1128/iai.63.2.456-466.1995 PMC173017

[28] Tongren JE, Drakeley CJ, McDonald SL, Reyburn HG, Manjurano A, Nkya WM, et al Target antigen, age, and duration of antigen exposure independently regulate immunoglobulin G subclass switching in malaria. Infect Immun. 2006;74(1):257–64. 10.1128/IAI.74.1.257-264.2006 16368979 PMC1346665

[29] França CT, He W-Q, Gruszczyk J, Lim NTY, Lin E, Kiniboro B, et al Plasmodium vivax Reticulocyte Binding Proteins Are Key Targets of Naturally Acquired Immunity in Young Papua New Guinean Children. PLOS Neglected Tropical Diseases. 2016;10(9):e0005014 10.1371/journal.pntd.0005014 27677183 PMC5038947

[30] Scopel KK, Fontes CJ, Ferreira MU, Braga EM. Factors associated with immunoglobulin G subclass polarization in naturally acquired antibodies to Plasmodium falciparum merozoite surface proteins: a cross-sectional survey in Brazilian Amazonia. Clinical and vaccine immunology: CVI. 2006;13(7):810–3. 10.1128/CVI.00095-06 16829621 PMC1489569

[31] Williams TN. Red blood cell defects and malaria. Molecular and biochemical parasitology. 2006;149(2):121–7. 10.1016/j.molbiopara.2006.05.007 16797741

[32] Amoako N, Asante KP, Adjei G, Awandare GA, Bimi L, Owusu-Agyei S. Associations between red cell polymorphisms and Plasmodium falciparum infection in the middle belt of Ghana. PLOS ONE. 2014;9(12):e112868 10.1371/journal.pone.0112868 25470251 PMC4254276

[33] Zimmerman PA, Ferreira MU, Howes RE, Mercereau-Puijalon O. Red blood cell polymorphism and susceptibility to Plasmodium vivax. Adv Parasitol. 2013;81:27–76. 10.1016/B978-0-12-407826-0.00002-3 23384621 PMC3728992

[34] Serjeantson SW, White BS, Bhatia K, Trent RJ. A 3.5 kb deletion in the glycophorin C gene accounts for the Gerbich-negative blood group in Melanesians. Immunol Cell Biol. 1994;72(1):23–7. 10.1038/icb.1994.4 8157284

[35] Rosenfield RE, Haber GV, Kissmeyer-Nielsen F, Jack JA, Sanger R, Race RR. Ge, a very common red-cell antigen. British journal of haematology. 1960;6:344–9. 13743453 10.1111/j.1365-2141.1960.tb06251.x

[36] Booth PB, McLoughlin K. The Gerbich blood group system, especially in Melanesians. Vox sanguinis. 1972;22(1):73–84. 5011657 10.1111/j.1423-0410.1972.tb03968.x

[37] Serjeantson SW. A selective advantage for the Gerbich-negative phenotype in malarious areas of Papua New Guinea. P N G Med J. 1989;32(1):5–9. 2750321

[38] Patel SS, Mehlotra RK, Kastens W, Mgone CS, Kazura JW, Zimmerman PA. The association of the glycophorin C exon 3 deletion with ovalocytosis and malaria susceptibility in the Wosera, Papua New Guinea. Blood. 2001;98(12):3489–91. 11719395 10.1182/blood.v98.12.3489

[39] Lin E, Tavul L, Michon P, Richards JS, Dabod E, Beeson JG, et al Minimal association of common red blood cell polymorphisms with Plasmodium falciparum infection and uncomplicated malaria in Papua New Guinean school children. The American journal of tropical medicine and hygiene. 2010;83(4):828–33. 10.4269/ajtmh.2010.09-0713 20889874 PMC2946751

[40] Koepfli C, Colborn KL, Kiniboro B, Lin E, Speed TP, Siba PM, et al A high force of plasmodium vivax blood-stage infection drives the rapid acquisition of immunity in papua new guinean children. PLOS Negl Trop Dis. 2013;7(9):e2403 10.1371/journal.pntd.0002403 24040428 PMC3764149

[41] Tavul L, Mueller I, Rare L, Lin E, Zimmerman PA, Reeder J, et al Glycophorin C delta(exon3) is not associated with protection against severe anaemia in Papua New Guinea. P N G Med J. 2008;51(3–4):149–54. 21061946

[42] Singh S, Pandey K, Chattopadhayay R, Yazdani SS, Lynn A, Bharadwaj A, et al Biochemical, biophysical, and functional characterization of bacterially expressed and refolded receptor binding domain of Plasmodium vivax duffy-binding protein. J Biol Chem. 2001;276(20):17111–6. 10.1074/jbc.M101531200 11279211

[43] Kellar KL, Kalwar RR, Dubois KA, Crouse D, Chafin WD, Kane BE. Multiplexed fluorescent bead-based immunoassays for quantitation of human cytokines in serum and culture supernatants. Cytometry. 2001;45(1):27–36. 11598944 10.1002/1097-0320(20010901)45:1<27::aid-cyto1141>3.0.co;2-i

[44] Bhardwaj R, Shakri AR, Hans D, Gupta P, Fernandez-Becerra C, Del Portillo HA, et al Production of recombinant PvDBPII, receptor binding domain of Plasmodium vivax Duffy binding protein, and evaluation of immunogenicity to identify an adjuvant formulation for vaccine development. Protein expression and purification. 2017;136:52–7. 10.1016/j.pep.2015.06.011 26578115

[45] He WQ, Shakri AR, Bhardwaj R, Franca CT, Stanisic DI, Healer J, et al Data from: Antibody Responses to Plasmodium vivax Duffy Binding and Erythrocyte Binding Proteins Predict Risk of Infection and are Associated with Protection from Clinical Malaria. Dryad Digital Repository. 10.1371/journal.pntd.0006987 PMC6400399 30768655

[46] Chen E, Salinas ND, Huang Y, Ntumngia F, Plasencia MD, Gross ML, et al Broadly neutralizing epitopes in the Plasmodium vivax vaccine candidate Duffy Binding Protein. Proc Natl Acad Sci U S A. 2016;113(22):6277–82. 10.1073/pnas.1600488113 27194724 PMC4896725

[47] Healer J, Thompson JK, Riglar DT, Wilson DW, Chiu Y-HC, Miura K, et al Vaccination with conserved regions of erythrocyte-binding antigens induces neutralizing antibodies against multiple strains of Plasmodium falciparum. PLOS ONE. 2013;8(9):e72504 10.1371/journal.pone.0072504 24039774 PMC3769340

[48] Rosanas-Urgell A, Lin E, Manning L, Rarau P, Laman M, Senn N, et al Reduced risk of Plasmodium vivax malaria in Papua New Guinean children with Southeast Asian ovalocytosis in two cohorts and a case-control study. PLoS Med. 2012;9(9):e1001305 10.1371/journal.pmed.1001305 22973182 PMC3433408

