# Supplementary figures and images for: Antibody responses to Plasmodium vivax Duffy binding and Erythrocyte binding proteins predict risk of infection and are associated with protection from clinical Malaria
Source: PLoS Negl Trop Dis. 2019 Feb 15;13(2):e0006987. doi: 10.1371/journal.pntd.0006987 (PMC6400399; doi:10.1371/journal.pntd.0006987)

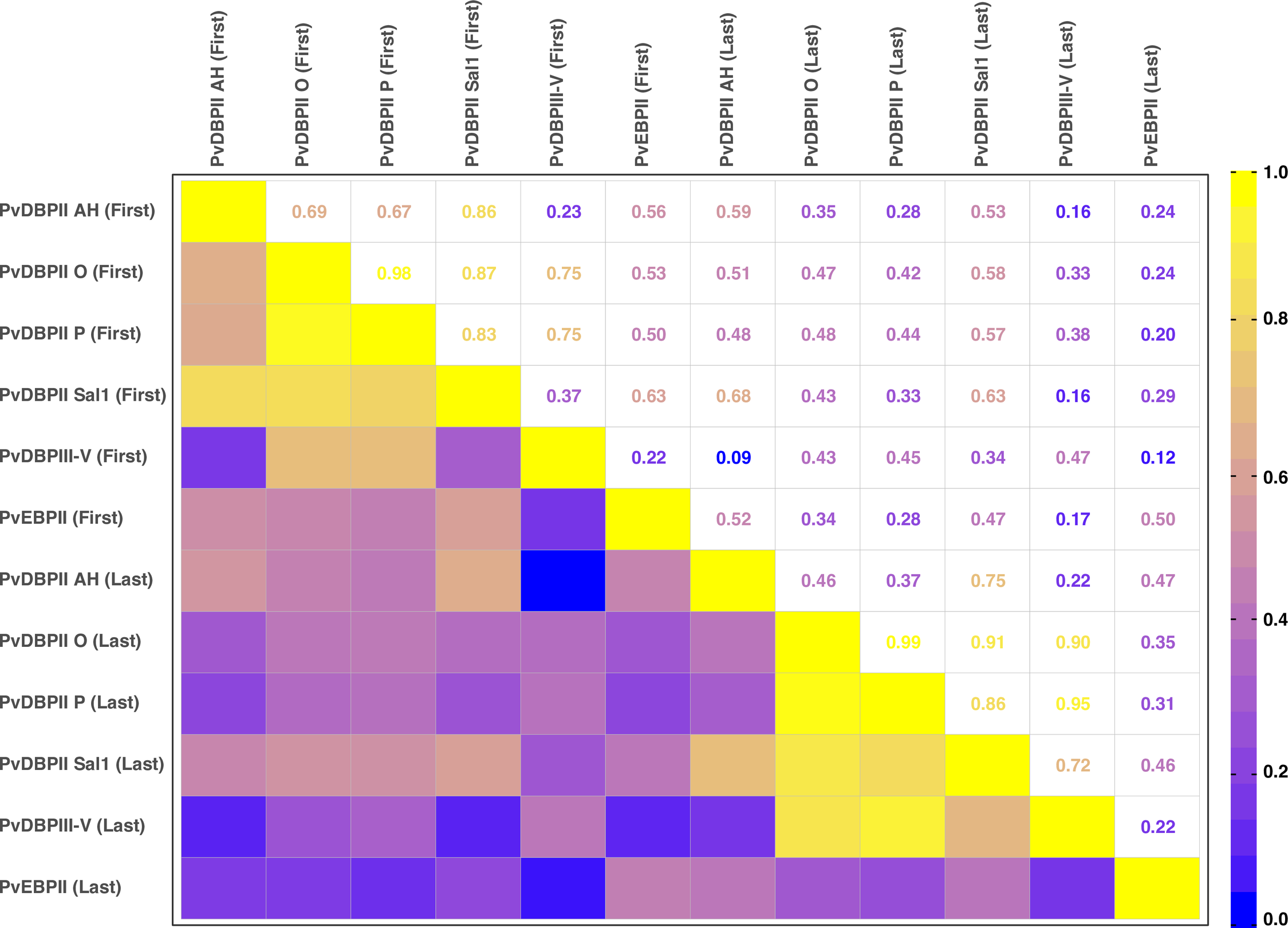

Supplement: S1 Fig — The heat map colors correspond to the Spearman’s correlation coefficient, ranging from 0 (no correlation, blue) to 1 (strong correlation, yellow). P<0.001–0.152. (TIF) [file pntd.0006987.s002.tif]

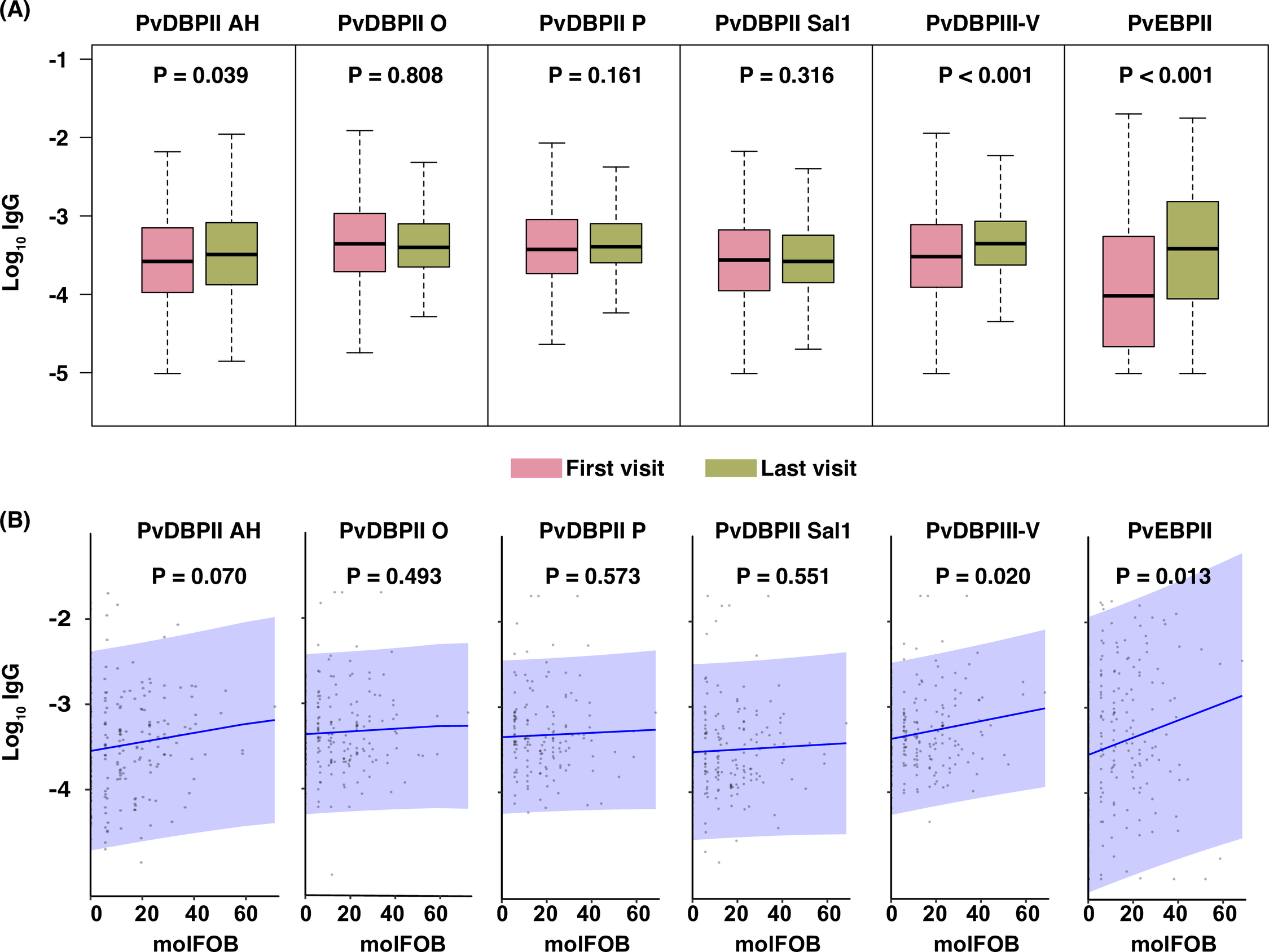

Supplement: S2 Fig — (A) The comparison of antibody levels between enrolment and end of the cohort study. Red represents the first visit and blue represents the last visit. P values were calculated using Wilcoxon signed-rank sum method. P values <0.05 were considered significant. (B) Association of antibody levels from the last visit of the study and the molecular force of blood stage infection (molFOB). The blue lines show the association between antibody responses and molFOB predicted by linear regression models. The shaded regions depict the variation in the data (95% prediction interval). X-axis: molFOB, y-axis: total IgG antibody responses for each antigen. P values are from general linear model. P values and were deemed significant if <0.05. (TIF) [file pntd.0006987.s003.tif]

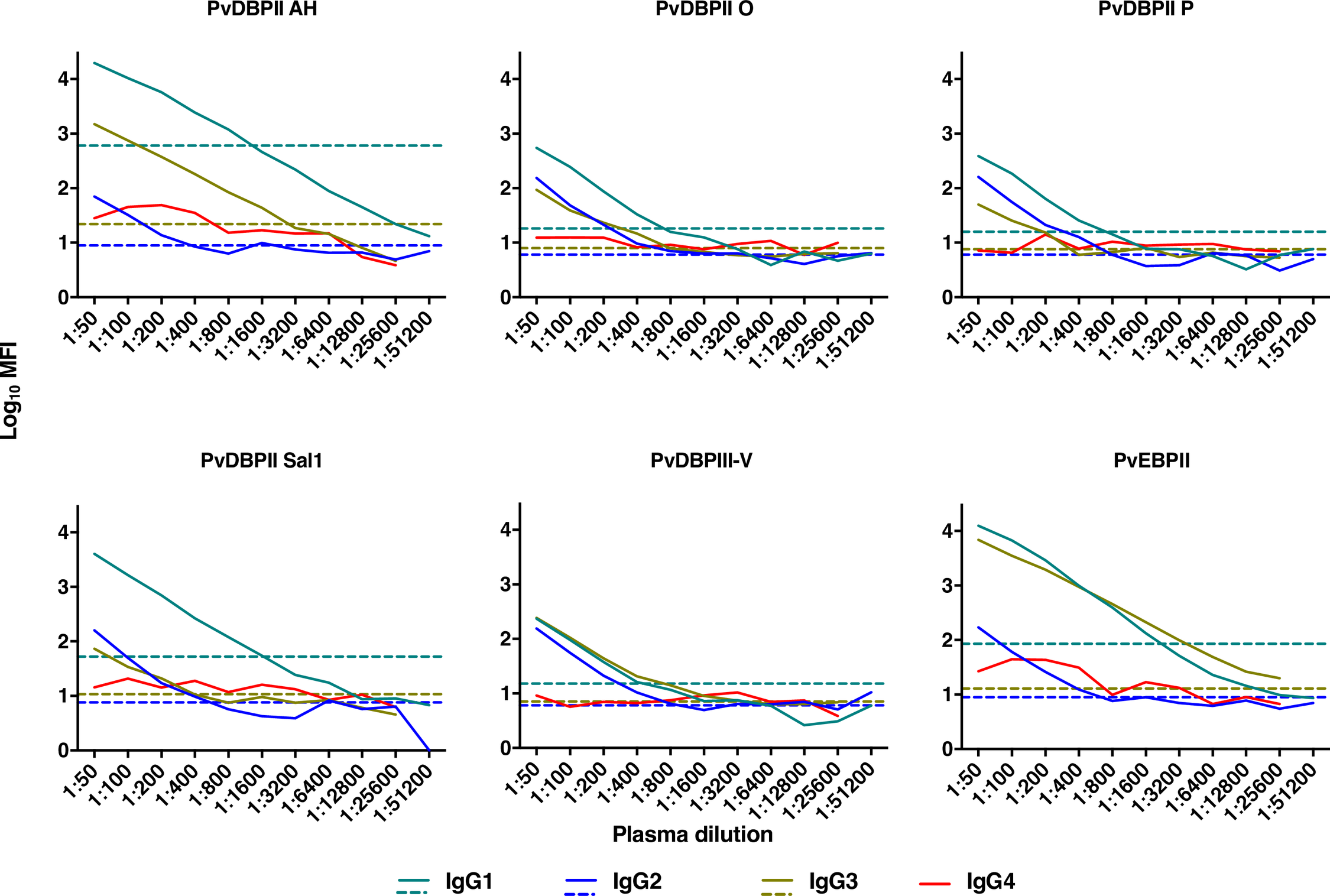

Supplement: S3 Fig — Antibody levels of crude mean fluorescence intensity (MFI) were log10 transformed. Solid lines represent antibody levels among adults in a two-fold serial dilution starting from 1/50. Only the median antibody levels among children for each subclass (IgG1, IgG2 and IgG3) were presented by dashed lines. (TIF) [file pntd.0006987.s004.tif]

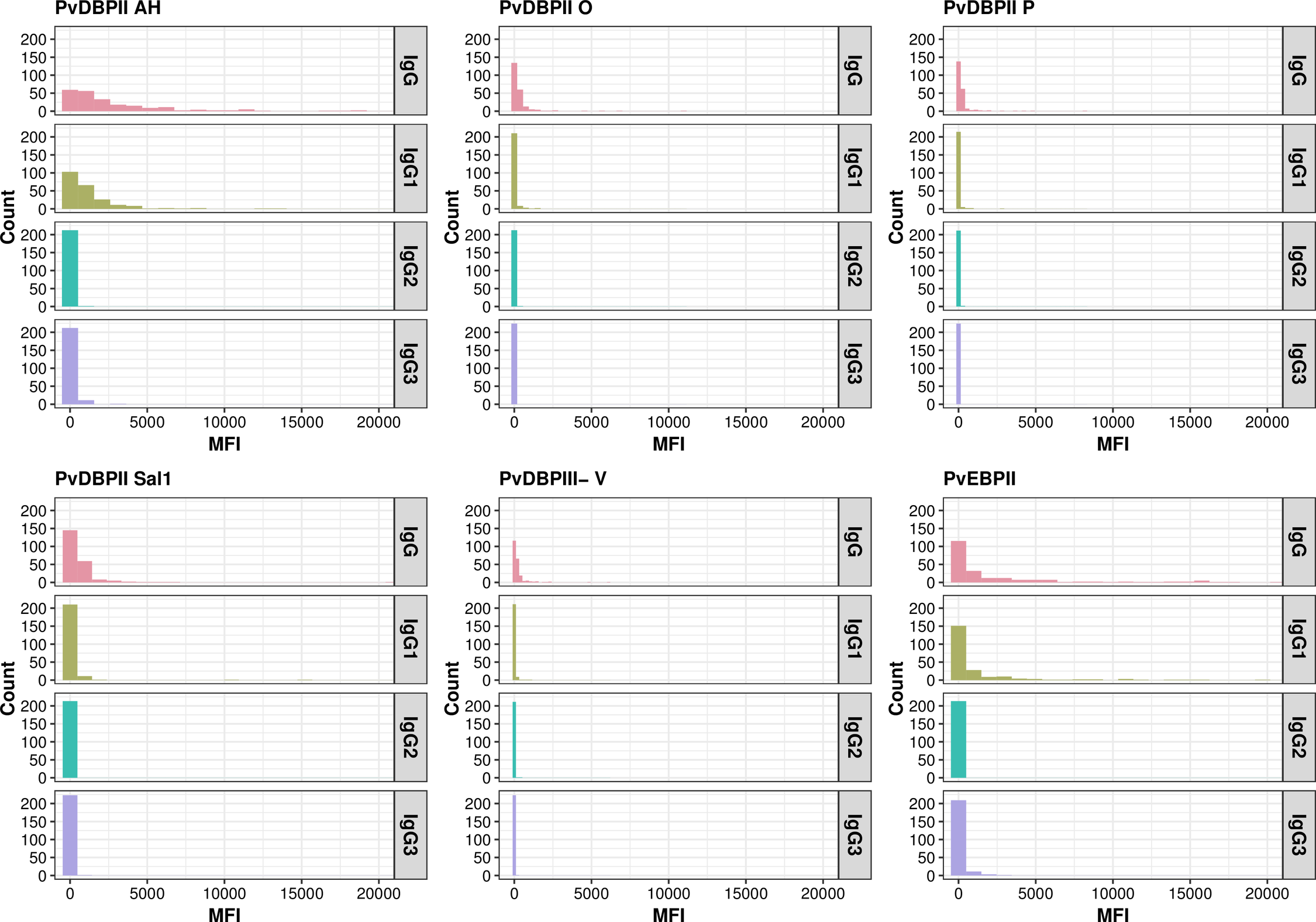

Supplement: S4 Fig — Antibody levels of crude mean fluorescence intensity (MFI) were shown in X axis and count of each level were represented in Y axis. Antibody levels of total IgG, IgG1, IgG2 and IgG3 were depicted in pink, yellow, blue, and light slate blue respectively. (TIF) [file pntd.0006987.s005.tif]
